# Tensile strength of nanocrystalline FeCoNi medium-entropy alloy fabricated using electrodeposition

**DOI:** 10.1038/s41598-022-16086-6

**Published:** 2022-07-15

**Authors:** Atsuya Watanabe, Takahisa Yamamoto, Yorinobu Takigawa

**Affiliations:** 1 Department of Materials Science, Osaka Prefecture University (OPU), 1-1 Gakuen-cho,Naka-ku, Sakai, Osaka 599-8531 Japan; 2 Department of Materials Design Innovation Engineering, Nagoya University, Furo-cho, Chikusa-ku, Nagoya, Aichi 464-8603 Japan; 3 Present Address: Department of Materials Science, Osaka Metropolitan University (OMU), 1-1 Gakuen-cho, Naka-ku, Sakai, Osaka 599-8531 Japan

**Keywords:** Mechanical properties, Metals and alloys, Electrocatalysis, Nanoscale materials

## Abstract

Crystal-grain refinement is one of the effective approaches to obtaining high-strength materials. A good strength/ductility balance has been reported in fine grains of high- and medium-entropy alloys. However, crystal-grain refinement at the nanometer scale has not been achieved yet. In this study, we used electrodeposition to fabricate 0.2-mm thick equiatomic FeCoNi medium-entropy alloys (MEAs) with 10-nm crystal grains. The nanocrystalline FeCoNi MEAs exhibit the maximum tensile strength of 1.6 GPa, which is the highest reported result to date.

## Introduction

High-entropy alloys (HEAs) and medium-entropy alloys (MEAs)^1,2^ have received attention in the last decade due to their promising properties, such as good strength/ductility balance^2,3^, wear resistance^4^, and corrosion resistance^5^. These alloys consist of three or more equiatomic elements, i.e., located at the center of a multicomponent phase diagram.

Crystal-grain refinement of HEAs/MEAs is an effective approach for developing high-strength materials. Similarly to other metallic materials, the strength of HEAs/MEAs depends on the crystal grain size as per the Hall–Petch (HP) relation:

1 $$\begin{aligned} H=H_0+kd^{-1/2} \end{aligned}$$

where *H* is the strength, $H_0$ is the frictional stress, *d* is the grain size, and *k* is a constant (HP slope)^6,7^. Previous works on HEAs/MEAs with micron order grains have demonstrated a large HP slope compared with conventional metallic materials, such as nickel^3,8^. Further, superior high-temperature stability of the grain size in HEAs/MEAs arising from sluggish diffusion expands the practical temperature range of conventional nanocrystalline metals^8–10^. However, the dependence of strength on the grain size has a peak, and the crystal-grain refinement reduces strength in the nanometer order region^11^. The peak grain size has been already investigated by experiment^10^ and simulation^12^ in CrMnFeCoNi HEAs and their subsystem MEAs, with the alloys with a grain size of $\sim$10 nm exhibiting the highest strength.

At the moment, one of the issues in the field is that crystal-grain refinement to nanometer order and fabrication as thick materials have not been achieved simultaneously^13^. One of the most general approaches to reducing the grain size on micron order is thermo-mechanical processing and severe plastic deformation; however, the ultimate grain size exceeds 0.1 µm due to recrystallization. On the other hand, nanocrystalline HEAs/MEAs have been typically obtained by vapor process as thin foils. Therefore, their mechanical properties have been examined by indentation testing, including nano-indentation testing, Vickers hardness testing, and more^10,13,14^. However, it is not easy to determine macroscopic mechanical properties including ductility with precision by these techniques. On the other hand, tensile tests typically require a thickness greater than 0.2 mm^15,16^. To clarify the deformation behaviors of nanocrystalline HEAs/MEAs, the fabrication process has to be compatible with both refinement of crystal grains to nanometer order and increasing thickness. Not to mention that increasing product size is important for practical applications.

In the past few years, there have been reports about the electrodeposition of nanocrystalline MEAs^10,17–19^. Electrodeposition is an electrochemical bottom-up process in the liquid phase that is potentially used for fabricating bulk materials compared with the vapor process. Moreover, the low-temperature process allows for the formation of metastable microstructures, such as nanocrystalline and supersaturated solid solutions^20–23^. In fact, some efforts in electrodeposition succeeded to fabricate nanocrystalline MEAs with grain sizes less than 10 nm.

In this study, we fabricated equiatomic FeCoNi MEAs with a 10 nm grain size and $\sim$0.2 mm thickness via electrodeposition in an aqueous solution. The samples exhibited the highest tensile strength among the FeCoNi MEAs reported to date. Insights on the relationship between mechanical properties and microstructure provide a strategy to develop electrodeposition for HEAs/MEAs that combine high strength and ductility.

## Results and discussion

### Microstructural characterization

**Figure 1:**
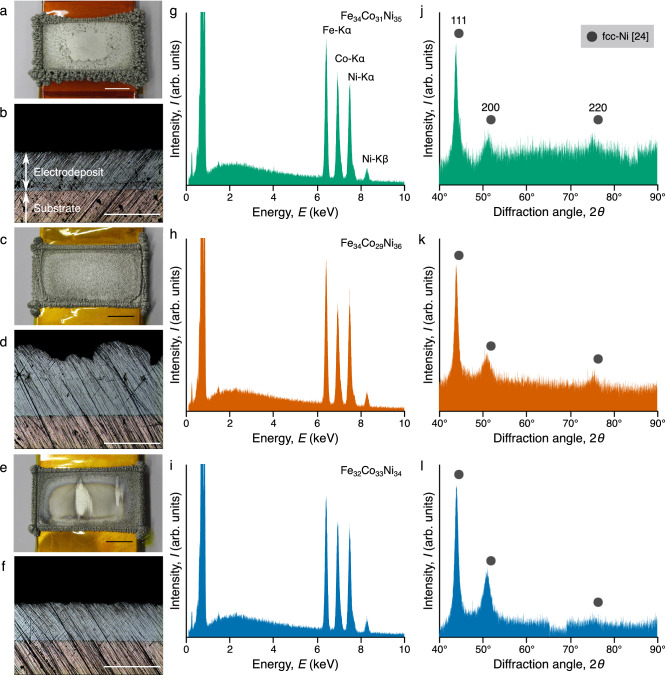
Appearance and microstructural analysis of the electrodeposited nanocrystalline FeCoNi MEAs. (**a**, **c**, and **e**) Surface appearance with a 10-mm scale bar, (**b**, **d**, and **f**) cross-section appearance with a 0.5-mm scale bar, (**g**, **h**, and **i**) EDX spectra, (**j**, **k**, and **l**) XRD profiles of samples A, B, and C, respectively.

Three platy samples (A, B, and C) with a minimum thickness of 0.2 mm were produced on the copper substrates in each 50-h electrodeposition process. The surface and cross-section appearances of the samples are shown in Fig. 1a–f, respectively. The samples had some morphological variations in surface structure.

The samples’ compositions were determined from their energy-dispersive X-ray (EDX) spectra, as shown in Fig. 1g–i. All the samples (A, B, and C) were identified as equiatomic FeCoNi MEAs (${\mathrm {Fe}}_{34}{\mathrm {Co}}_{31}{\mathrm {Ni}}_{35}$, ${\mathrm {Fe}}_{34}{\mathrm {Co}}_{29}{\mathrm {Ni}}_{35}$, ${\mathrm {Fe}}_{32}{\mathrm {Co}}_{33}{\mathrm {Ni}}_{34}$, respectively). The samples had no significant shifts in composition associated with continuous electrodeposition.

The X-ray diffraction (XRD) profiles of the samples are shown in Fig. 1j–l. The appeared diffraction peaks indicate a face-centered cubic (fcc) structure^24^. It is notable that the later a sample was prepared, the stronger the intensity of 200 diffraction peaks. Furthermore, the crystallite size $\Lambda$ of $\sim$10 nm was derived from the width of the 111 diffraction peaks using the Scherrer equation^25^:

2 $$\begin{aligned} \Lambda =\frac{K\lambda }{B\cos \theta } \end{aligned}$$

where $\lambda$ is the X-ray wavelength, *B* is the full width at half maximum, and $\theta$ is half of the diffraction angle. As the shape factor, $K=0.9$ was used.

**Figure 2:**
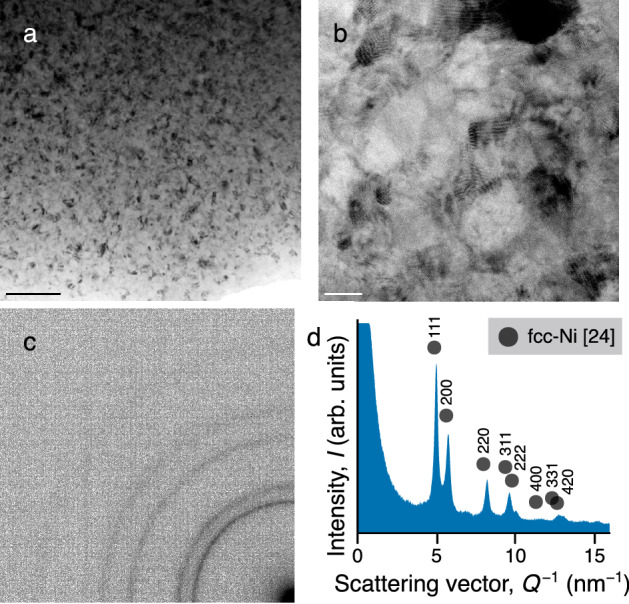
TEM observation of the electrodeposited nanocrystalline FeCoNi MEAs. (**a**) BF-TEM image in a wide area and (**b**) BF-STEM image in high magnification of sample C. The scale bars represent 100 nm and 10 nm, respectively. (**c**) Electron diffraction pattern and (**d**) intensity distribution.

Figure 2a and b show the representative bright-field transmission electron microscopy (BF-TEM) image and bright-field scanning transmission electron microscopy (BF-STEM) image, respectively. Nanocrystals of $\sim$10 nm were observed in accordance with the crystallite size calculated from the Scherrer equation. The electron diffraction pattern (Fig. 2c) shows some continuous Debye-Scherrer rings. As shown in Fig. 2d, the rings are indexed as attributed to an fcc structure. All of them confirm the successful fabrication of thick nanocrystalline FeCoNi MEAs by electrodeposition.

### Mechanical characterization

**Figure 3:**
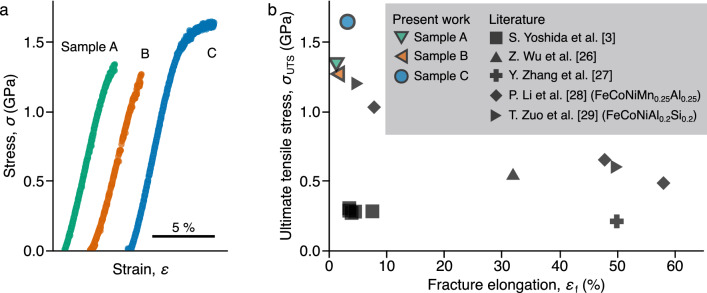
Mechanical properties of the electrodeposited nanocrystalline FeCoNi MEAs. (**a**) Stress–strain curves of samples A, B, and C. (**b**) UTS and fracture elongation in this work compared with the literature^3,26–29^.

Tensile behaviors were examined using specimens cut out from the electrodeposited samples. The stress–strain curves are shown in Fig. 3a. Samples A, B, and C exhibited ultimate tensile stresses (UTSs) of 1.3 GPa, 1.3 GPa, and 1.6 GPa and fracture elongations of 1.4%, 1.6%, and 3.3% respectively. All three samples exhibited high tensile strength, although the fracture elongation varied among the samples. The ultimate thickness of the tensile specimens post polishing slightly differed. However, the fracture elongation did not correlate to the ultimate thickness of the tensile specimens.

Figure 3b presents the strength and fracture elongation of the three samples compared with the literature data^3,26–29^. The present samples exhibit higher UTSs than the reported FeCoNi, and FeCoNi-based MEAs containing small amounts of additional elements. Also, the ductility of the samples hardly decrease despite the significant increase in the strength.

Despite the dispersion in tensile strength, the as-deposited A, B, and C samples exhibited almost the same Vickers hardness: 506 HV, 497 HV, and 513 HV, respectively. In the case of sample C, the proportion of hardness to UTS ($H_{\mathrm {V}}/\sigma _{\mathrm {UTS}}$) was 3.1. On the other hand, samples A and B exhibited higher proportions: 3.7 and 3.9, respectively. It is known that Vickers hardness ($H_{\mathrm {V}}$) is proportional to UTS ($\sigma _{\mathrm {UTS}}$). The proportion is typically $\sim 3.0$^30^; the increasing proportion implies a premature fracture caused by flaws^15,16,31^. It is considered that the difference in tensile strength between the samples results from a premature failure ascribed to uneven ductility. Hence, the tensile strength of 1.6 GPa demonstrated by sample C is very intrinsic for a FeCoNi MEA with a grain size of 10 nm.

The reason for the premature failure occurrence in the samples is also worth discussing. In electrodeposited nickel, the 200 orientation index ($N_{200}$) suggested to be correlating with ductility^32,33^ is calculated as follows:

3 $$\begin{aligned} N_{200}=\frac{I_{200}}{I_{111}+I_{200}+I_{220}} \end{aligned}$$

where $I_{hkl}$ is the intensity of the *hkl* diffraction peak in the XRD profile. This correlation has been considered to be caused by the generated hydrogen inhibiting the deposit growth. The process-derived flaws often cause a premature fracture in nanocrystalline materials^34^. In this work, the 200 orientation indexes of samples A, B, and C were found to be 0.18, 0.13, and 0.25, respectively. The ductility of the samples depends on the 200 orientation index, the same way as in electrodeposited nickel. This fact implies that controlling the 200 orientation index is one of the ways to improve the ductility of electrodeposited MEAs. It is not clear why the crystal orientation changes between the samples which were fabricated earlier and later; however, it may be caused by additive agents in aqueous solutions, such as brighteners and surfactants, because these chemicals are consumed during the electrodeposition^35,36^.

**Figure 4:**
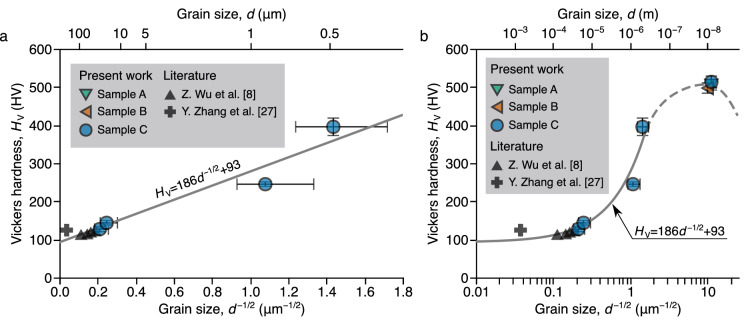
Hall–Petch relationship for the FeCoNi MEAs. Vickers hardness of the FeCoNi MEAs previously reported^8,27^ and fabricated in this work on the Hall–Petch relationship presented in (**a**) linear and (**b**) log scales for grain size. The error bars present the standard deviation for measured grain size and hardness.

Furthermore, we investigated the distribution of the nanocrystalline FeCoNi MEAs on the HP relationship. The electrodeposited nanocrystalline samples were annealed at 500–800 °C for 1 h. The crystal-grain distributions of the annealed samples were measured by the electron backscatter diffraction (EBSD) technique. The obtained inverse pole figure maps and crystal-grain size distribution are shown in Supplementary Fig. 1. Figure 4 presents the hardness versus grain size obtained in this study compared with literature data. The error bars represent the standard deviation in grain size and hardness. In the region of grain size above 0.5 μm, the data points obtained in this study and the literature^8,27^ are on a line, whose function is determined as:

4 $$\begin{aligned} H_{\mathrm {V}}=186d^{-1/2}+93. \end{aligned}$$

The coefficient of determination ${R_1}^2$ was 0.939. This relationship corresponds reasonably well with the one already suggested ($H_{\mathrm {V}}=131.1d^{-1/2}+97.3$)^8^. On the other hand, the as-deposited samples with $\sim$10 nm grains exhibited higher hardness than the annealed samples with $d\sim$0.5 µm. However, the strengthening was lower than extrapolated from Eq. (4).

The inverse HP effect should be considered to discuss the inhibition of strengthening by crystal-grain refinement at the nanometer scale^11,37,38^, as described in the introduction section. The fraction of grain boundaries increases with the refinement of crystal grains. As a consequence of extreme grain size refinement, the grain boundary deformation, such as grain boundary sliding, migration, and rotation, becomes dominant compared with the movement of the dislocations in the grain interior^39,40^. Accordingly, the alloy strength does not increase but rather decreases with the grain size refinement. The lower strengthening than extrapolated from the HP relationship indicates approaching the region with dominant grain boundary deformation and supports the peak strength at a $\sim$10 nm grain size. Further, it is also considered that once the grain boundary deformation mechanism is initiated, the strengthening mechanisms in HEAs/MEAs considered previously, such as the interaction between slipping dislocations and lattice distortion, stop working. Therefore, the conventional model of HEAs/MEAs might not apply to the deformation behaviors in nanocrystalline HEAs/MEAs. Further development is needed in the fabrication process to clarify the effects of alloying elements on the deformation of nanocrystalline HEAs/MEAs.

It is also important to clarify the detailed dependence of strength on the grain size near the peak. However, controlling grain size through heat treatment is not easy because the abnormal grain growth causes leaps in grain size from nanometer order to micrometer order^8,10^. Further, the dependence of strength on the grain size will be detailed by controlling grain size via consideration of electrodeposition parameters, such as additive agents and current density.

In summary, equiatomic FeCoNi MEAs with a thickness of $\sim$0.2 mm and a grain size of $\sim$10 nm were fabricated via electrodeposition in this study. In tensile tests, the nanocrystalline FeCoNi MEAs exhibit a tensile strength of up to $\sim$1.6 GPa. This is the highest strength reported as UTS of a FeCoNi MEA. The lower strengthening with crystal-grain refinement than estimated by the HP relationship for micron-scale grains implies the start of grain boundary deformation in the FeCoNi MEA with a 10 nm grain size.

## Methods

### Alloy sample preparation

Three nanocrystalline FeCoNi MEAs were sequentially electrodeposited for $\sim$50 h each in an aqueous solution (5 L) comprising 600 mol m^−3^ boric acid, 50 mol m^−3^ hydroxylammonium chloride, 15 mol m^−3^ saccharin sodium dihydrate, 5.0 mol m^−3^ sodium dodecyl sulfate, 500 mol m^−3^ nickel(II) sulfamate tetrahydrate, 80 mol m^−3^ cobalt(II) sulfate hyptahydrate, and 220 mol m^−3^ iron(II) sulfate hyptahydrate. All chemicals were supplied by FUJIFILM Wako Pure Chemical (Osaka, Japan). Copper substrates covered with polyimide tape except for a $21 \times 40$ mm^2^ area were employed as the cathode, and two Pt/Ti rods were used as the anodes. The solution temperature was kept at 55 ± 4 °C during the electrodeposition. The total cathode current density was set to 50 mA cm^−2^. The details of electrodeposition equipment are described elsewhere^33^.

### Microstructural and mechanical characterization

Dog-born shaped tensile test specimens with a gauge length of 10.00 mm and a gause width of 2.00 mm were cut out by electrical-discharge machining (EDM). The cut section was then polished with SiC paper, and the specimens were observed on an ECLIPSE MA100 optical microscope (NIKON, Japan).

Offcuts of the specimens were used for the microstructural characterization and Vickers hardness test. The samples were cut out followed by mechanical polishing with SiC paper and then finished to a mirror surface by buff polishing with 1 μm diamond powder. The samples for EBSD analysis were additionally finished by buff polishing with colloidal silica. To increase the grain size, some samples were annealed at 500 °C, 600 °C, 700 °C, and 800 °C, respectively, for 1 h in a muffle furnace in an air atmosphere, following mechanical polishing to remove the copper substrate.

A QUANTAX EDX device with an XFlash 6–10 detector (Bruker, USA) was operated on the SU8010 SEM (Hitachi High-Tech, Japan) at a 15 kV acceleration voltage. XRD profiles were acquired using an Ultima IV system with a D/teX Ultra detector (Rigaku, Japan) working at a 40 kV acceleration voltage with $\hbox {Cu-K}\alpha$ radiation at the 0.1542 nm wavelength. EBSD measurements used an MSC-2200 EBSD detector (TSL Solutions, Japan) attached to a JSM-7001F SEM (JEOL, Japan) operating at a 15 kV acceleration voltage. The collected data was analyzed using OIM analysis ver. 5.31 (TSL Solutions). For grain size determination, data points with a confidence index less than 0.2 and grain size less than five times of step size were omitted.

The microstructure of the as-deposited samples was observed using a JEM-2100Plus TEM (JEOL) and a JEM-ARM200F spherical aberration corrected STEM (JEOL) at a 200 kV acceleration voltage. TEM samples were thinned down to 0.1 mm by mechanical polishing followed by buff polishing. The samples were then finished using argon-ion milling equipment (model 691, Gatan, USA) at a 2.5 kV acceleration voltage. The intensity distribution on the electron diffraction pattern was analyzed using ProcessDiffraction ver. 8.7.1Q^41^.

Before the tensile testing, the specimens were mechanically polished to remove the copper substrate and the EDM-damaged layer. Tensile testing was conducted at a 1.0 × 10^−3^ s^−1^ strain rate and 20 °C using a 8874 tabletop fatigue testing system (Instron, USA) with a custom-made inset type jig. Vickers hardness test employed a DMH-2 tester (Matsuzawa Co., Ltd, Japan) with a 100 gf load at 10 s and 20 °C. The fracture elongation was derived from the changes in the gauge length after the test.

## Supplementary Information

Supplementary Figure S1.

## Data Availability

The data that support the findings of the present work are available from the corresponding author on reasonable request.
