# Supplementary material for: Tensile strength of nanocrystalline FeCoNi medium-entropy alloy fabricated using electrodeposition
Source: Sci Rep. 2022 Jul 15;12:12076. doi: 10.1038/s41598-022-16086-6 (PMC9287447; doi:10.1038/s41598-022-16086-6)
Supplement: Supplementary file 1 — Supplementary Figure S1. [file 41598_2022_16086_MOESM1_ESM.pdf]

(Supplementary material)
Tensile strength of nanocrystalline FeCoNi medium-entropy alloy fabricated using electrodeposition

Atsuya Watanabe[1,3,*], Takahisa Yamamoto[2], Yorinobu Takigawa[1,3]

[1] Department of Materials Science, Osaka Prefecture University (OPU), 1-1 Gakuen-cho, Naka-ku, Sakai, Osaka 599-8531, Japan

[2] Department of Materials Design Innovation Engineering, Nagoya University, Furo-cho, Chikusa-ku, Nagoya, Aichi 464-8603, Japan

[3] Present address: Department of Materials Science, Osaka Metropolitan University (OMU), 1-1 Gakuen-cho, Naka-ku, Sakai, Osaka 599-8531, Japan

[*] watanabe@osakafu-u.net

Contents

Supplementary Figure 1.

Crystal grain distribution of annealed sample C. 2

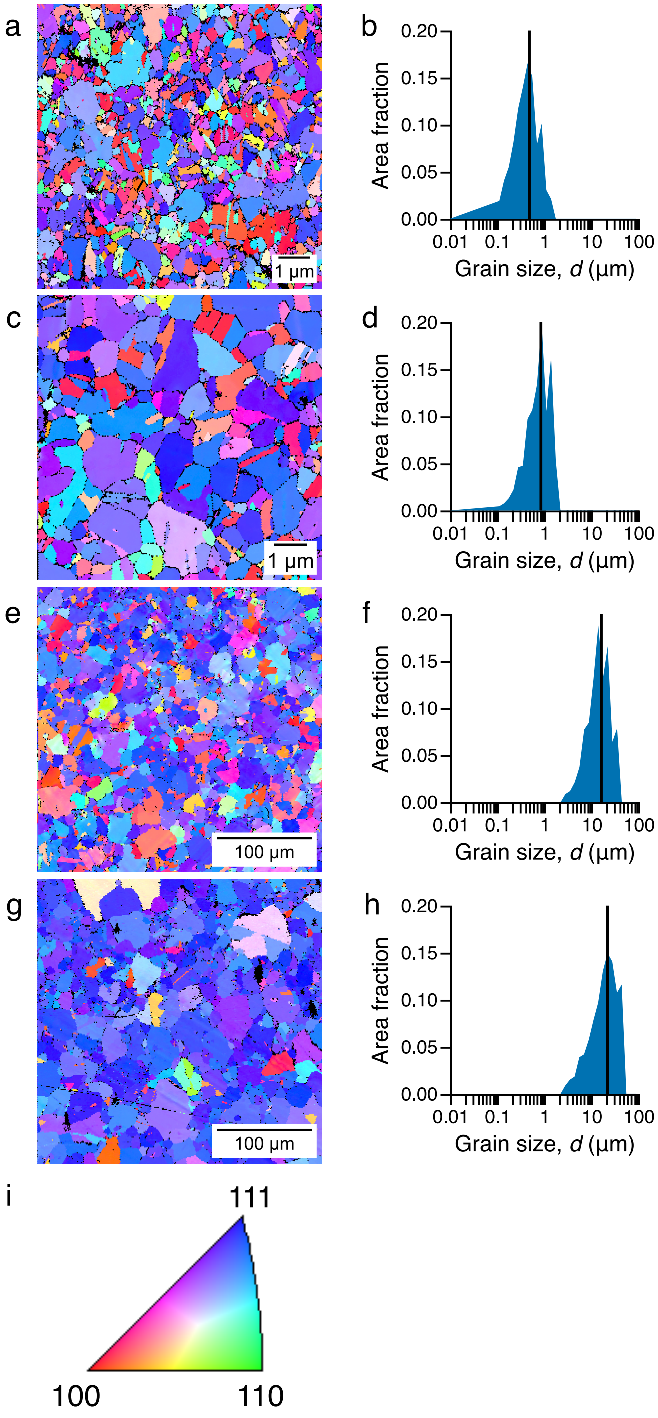

Supplementary Figure 1: Crystal grain distribution of annealed sample C. (**a, c, e, g**) Inverse pole figure maps collected in the samples annealed at (**a**) 500 °C, (**c**) 600 °C, (**e**) 700 °C, and (**g**) 800 °C, respectively, for 1 h. (**b, d, f, h**) Crystal grain size distribution in the each inverse pole figure maps. (**i**) The color legend.
